# Strengthening one-health in the Caribbean: a critical priority for the Americas

**DOI:** 10.3389/fvets.2026.1809427

**Published:** 2026-05-05

**Authors:** Luis Pablo Hervé-Claude, Juan Pablo Villanueva Cabezas, María José Navarrete-Talloni, Christa Gallagher

**Affiliations:** 1 Department of Clinical Sciences, Lewyt College of Veterinary Medicine, Long Island University - Post Campus, Brookville, NY, United States; 2 Department of Biomedical Sciences, Ross University School of Veterinary Medicine, Basseterre, Saint Kitts and Nevis; 3 Facultad de Medicina Veterinaria, Universidad San Sebastián, Concepción, Chile

**Keywords:** African Swine Fever (ASF), Avian Influenza (AI), heartwater (cowdriosis), New World Screwworm, rabies

The Caribbean region is frequently overlooked in discussions of global animal health. It comprises numerous island countries and territories situated along major trade and tourist routes linking Europe, North America, and Asia. Despite its strategic location, the Caribbean is characterized by significant fragmentation across physical, cultural, and political dimensions. The region faces challenges such as inadequate infrastructure, limited essential services, and insufficient regional coordination, which undermine effective responses to health threats. Consequently, the Caribbean remains especially susceptible to emerging animal, zoonotic, and vector-borne diseases.

**Figure 1:**
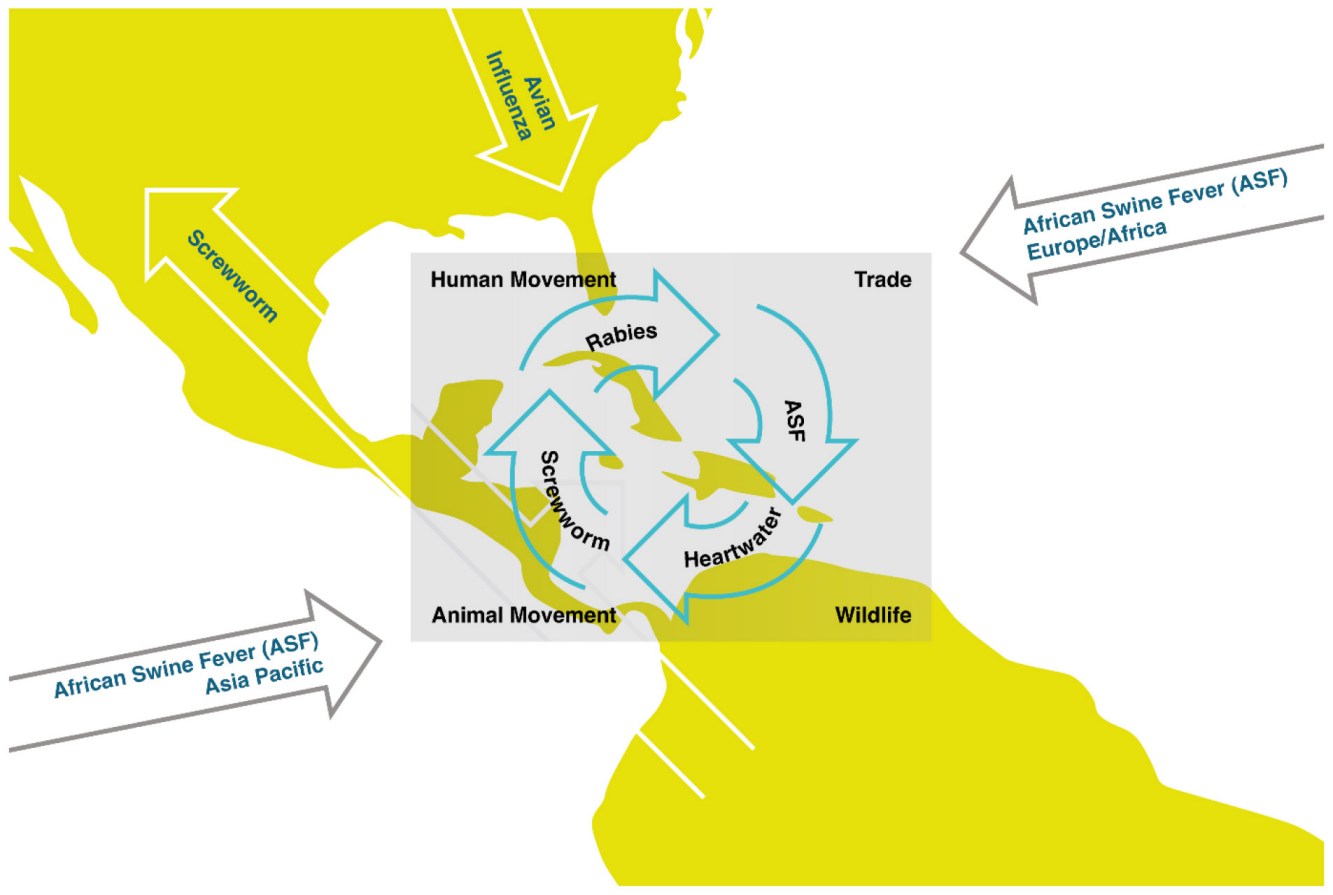
Exemplary key animal diseases and drivers in the Caribbean region. This figure was designed by LP Hervé-Claude.

Various diseases illustrate the issue (Figure 1). African Swine Fever (ASF) gained a foothold in the Caribbean, in the Dominican Republic and Haiti in 2021, becoming endemic on the island of La Hispaniola and threatening the entirety of the Americas through the movement of live animals, products and tourism (1). The zoonotic disease Screwworm is spreading across Central and North America, including Mexico, and threatens to spread further in the Caribbean beyond the known endemic islands of Jamaica, Cuba and Hispaniola through controlled and uncontrolled animal and human movements (2). Rabies is present in multiple territories, including Cuba, Grenada, the Dominican Republic and Puerto Rico which threatens humans, dogs, wildlife and livestock (3). H5 and H7 Avian Influenzas have caused significant outbreaks in North and South America, closely associated with the Caribbean region as migrating birds use the Atlantic & Mississippi flyways, often stopping in Caribbean islands which may facilitate incursion and spread of the virus in the region (4). Finally, Heartwater, a cattle tick-borne disease present only in the Caribbean islands of Guadeloupe, Marie-Galante, and Antigua, outside its endemic range in Africa, threatens the Americas' animal health because it remains uncontrolled due to the absence of regional coordination. Movement of infected livestock could transmit Heartwater to other islands as viable tick vectors exist in the region (5, 6). Although economic consequences have not been calculated for the smaller West Indies territories, these are important based on estimations for the USA, with potential economic impact of billions of USD if African Swine Fever or New World Screwworm is reintroduced (2, 7).

The intricacies produced by geopolitics lead to disjointed, reactive responses which are inadequate and unsustainable in the face of health threats. A One-Health approach, involving key stakeholders, multiple donors and a central coordinating entity, should be developed to create common key strategies that could reduce the risk and burden of these and other future animal health / zoonotic threats (8). Education at multiple levels (children, farmers, traders, etc.) coupled with the use of known biosecurity measures could make a difference in alleviating disease risks within this significant area.

The continuous systematic neglect of the Caribbean region directly threatens the Americas ecosystems and well beyond the Caribbean, as it acts as a potential reservoir for undesired and globally threatening diseases (Figure 1). The scientific and political communities must do better to promptly address the health security of Caribbean region and other susceptible populations abroad.

Nevertheless, some intergovernmental institutions have risen to the challenge. Among these CaribVet (9) is providing a platform to exchange ideas, functional working groups (e.g. Swine disease, Avian diseases, Veterinary Public Health, among others) and yearly Assemblies to accelerate regional collaboration and international research. In parallel, CAHFSA (10) works in the enhancement of regional agricultural health and food safety through the application of SPS (Sanitary and Phytosanitary) measures in the region. These, among other institutions, in partnership with international & national agencies (from France, the European Union, OIRSA, The United States of America, etc.) have materialized relevant project on One-Health, agricultural resilience, disaster preparedness, etc., with tangible results, being these examples of the importance of investing in Animal Health in a semi-structured way in the region.

Recently a group of six Caribbean island nations received a multi-million Pandemic Fund to bolster prevention, preparedness and response to emerging health threats, with a One-Health approach (11). This adds to the Cooperative Program in Research and Technology for the Northern Region, (12) grants of 2023 and 2024, with focus on Classical and African Swine fever (12) and USDA-APHIS funds mitigating the risk of ASF incursions into the US supporting the Caribbean region (13). A clear investment trend can be seen in the last 4 years, and this is surely going to reduce the information and capacity gap for the Eastern Caribbean.

The Caribbean region is facing important threats for animal and human health now. Urgent actions are needed. Although there are growing initiatives in the region, those need to be strengthened and expanded, to include all territories and embrace the geopolitical diversity of the Caribbean region. If no territory is left behind, regional growth and stability can be fostered. Strong political networking will be needed to include them all, as these range from overseas territories to independent nations, all with their own identities, complexities and structures. The immense richness of the Caribbean needs to be addressed if we want to succeed in promoting One-Health to the Caribbean region and the Americas.
